# Factors associated with lower gait speed among the elderly living in a developing country: a cross-sectional population-based study

**DOI:** 10.1186/s12877-015-0031-2

**Published:** 2015-04-01

**Authors:** Telma de Almeida Busch, Yeda Aparecida Duarte, Daniella Pires Nunes, Maria Lucia Lebrão, Michel Satya Naslavsky, Anelise dos Santos Rodrigues, Edson Amaro

**Affiliations:** Hospital Israelita Albert Einstein, Av. Albert Einstein, 670, São Paulo, SP Brazil; Department of Nursing, School of Nursing, University of São Paulo, São Paulo, SP Brazil; Department of Epidemiology, School of Public Health, University of São Paulo, São Paulo, SP Brazil; Human Genome Research Center, University of São Paulo, São Paulo, SP Brazil; Department of Radiology, University of São Paulo, São Paulo, SP Brazil

**Keywords:** Gait, Aged, Older adults, Walking speed

## Abstract

**Background:**

Among community-dwelling older adults, mean values for gait speed vary substantially depending not only on the population studied, but also on the methodology used. Despite the large number of studies published in developed countries, there are few population-based studies in developing countries with socioeconomic inequality and different health conditions, and this is the first study with a representative sample of population. To explore this, the association of lower gait speed with sociodemographic, anthropometric factors, mental status and physical health was incorporated participants’ weight (main weight) in the analysis of population of community-dwelling older adults living in a developing country.

**Methods:**

This was a cross-sectional population based on a sample of 1112 older adults aged 60 years and over from Health, Wellbeing and Aging Study cohort 2010. Usual gait speed (s) to walk 3 meters was stratified by sex and height into quartiles. Multiple regression analysis was performed to investigate the independent effect of each factor associated with a slower usual gait speed.

**Results:**

The average walking speed of the elderly was 0.81 m/s – 0.78 m/s among women and 0.86 m/s among men. In the final model, the factors associated with lower gait speed were age (OR = 3.56), literacy (OR = 3.20), difficulty in one or more IADL (OR = 2.74), presence of cardiovascular disease (OR = 2.15) and sedentarism. When we consider the 50% slower, we can add the variables handgrip strength, and the presence of COPD.

**Conclusions:**

Gait speed is a clinical marker and an important measure of functional capacity among the elderly. Our findings suggest that lower walking speed is associated with age, education, but especially with modifiable factors such as impairment of IADL, physical inactivity and cardiovascular disease. These results reinforce how important it is for the elderly to remain active and healthy.

## Background

Population aging is no longer a privilege of developed countries. Brazil has over 15 million elderly people (65 years or over) according to official census (IBGE), which corresponds to around 7.6% of the total population [1].

The great challenge of aging is to maintain one’s functional capacity, an individual’s ability to independently carry out activities deemed essential. There are several insidious and silent changes that occur with aging, making the distinction between senescence and senility very difficult [2]. The decline in physical performance is inevitable, and gait speed is considered a global indicator of functional mobility [3]. Reduced speed occurs with age [4,5] even among the healthy elderly [6], and it has a significant impact on one’s health and quality of life [6,7].

The change in gait speed is associated with physiological factors [7], behavioural factors [2], and the presence of diseases [8]. It may also increase the risk of falling [9] and result in disability, hospitalization [10-14], and death [15]. Reduced speed is associated with the risk of poor health-related outcomes. It is a component of phenotypic fragility [16], and also a clinical measure of functional assessment [10,17].

In spite of the fact that many studies have provided insights into the association between gait speed and all the factors mentioned above, only healthy elderly subjects were included [18,19], or potential confounders were not considered [19,20]. These associations were not consistent across studies and few investigations have been conducted with larger community-based, and this is the first one with a random weighted sample of population carried out a two-step sampling procedure with probability proportional to size using census tracts with replacement.

The objective of our study was to identify the factors associated with a lower gait speed in a representative sample of community-dwelling older adults in a developing country with many socioeconomic inequalities.

## Methods

### Study design

This study is part of the SABE (*Saúde, Bem Estar e Envelhecimento* / Health, Well-Being and Aging) Study. The SABE Study started in 2000 as a multicentre project coordinated by the Pan-American Health Organization, and it has been conducted in seven countries in Latin America and Caribbean. In Brazil, the study has been conducted in the city of São Paulo, selected through the multiple-stage sampling of census regions and it has become a longitudinal study. Survey that included 2.143 elderly individuals aged 60 or older in the city of São Paulo. A new wave has been performed in 2006 in the São Paulo initiative, and in this city the study became longitudinal. 1115 elderly subjects were reinterviewed (cohort A06) and a new cohort (B06) was added, the elderly aged 60–64 (n = 298), totalling 1413 individuals. In 2010, a third wave was performed, 989 elderly subjects (cohorts A, n = 748 and B, n = 242) were reinterviewed and a new cohort (C) of the elderly aged 60–64 (n = 355) was added. For this study, the sample was formed by wave 2010, cohorts A, B and C, totalling 1.345 elderly subjects (60 years and over). A detailed description of the methodology used can be found in [21].

**Figure 1:**
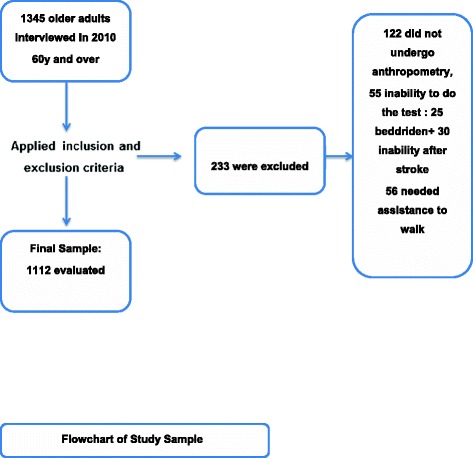
**Flowchart of Study Sample.**

For this study, individuals who were unable to perform the specific functional test (bedridden elderly) were excluded (n = 159), as well as subjects with neurological and/ or orthopaedic diseases, or those who used assistive devices in walking, or those with motor impairment resulted from stroke or with any pathological factors that might interfere with the gait speed(n = 74). The final sample comprised 1112 individuals. Figure 1 (Flowchart). This study received approval from the Human Research Ethics Committee of (IIEPAE) the Institute of Education and Research Albert Einstein (Brazil), protocol number 1360–11. Written informed consent was obtained from the subjects at the time of the interview.

### Measures and instruments

All data were collected at the participants’ homes by trained research assistants, and they included an interviewer-administered structured questionnaire with items on socioeconomic variables, general health and living conditions, as well as a set of anthropometric measures. The dependent variable in the present study was gait speed. The subject was instructed to walk three meters on a straight line marked on the ground at their usual speed, and thus the obtained time (in seconds) would be counted by using a hand-held stopwatch [22]. Two measurements were made and used for this study. The lowest walking speed (m/s) was considered. Walking speed was calculated by dividing the distance by the time it takes to cover a distance.

The following were the independent variables: 1) socio-demographic variables (age, sex, ethnicity, education, conjugal situation). Two reasons justify why cut-off points of education were set at 1-3years, 4–7 and 8 and more. One of them is that 21.0% of the elderly have never attended school, and 46.4% were younger than 4 years of schooling [21]. In addition, other studies have also used the same cut-off points [23,24]; 2) anthropometric variables: weight, height, body mass index (BMI). The classification of nutritional status followed the recommendations of the Pan American Health Organization: [25] 3) general health factors (number of self-reported chronic diseases (hypertension, diabetes mellitus, cardiovascular disease, cerebrovascular disease, chronic obstructive pulmonary disease (COPD), arthritis, osteoporosis), self-perceived general health (good/very good, regular or poor/very poor), and physical activity were evaluated by self-reported physical activity using the International Physical Activity Questionnaire (IPAQ) short form 8. This questionnaire determines the level of physical activity and it has been validated in a sample of the Brazilian population [26]. The practice of physical activity was assessed by the number of minutes spent per week to carry out the activities. This included walking or anything activity. The older adults who devoted 150 minutes weekly to perform moderate physical activities, or 75 minutes to vigorous physical activity, or an equivalent combination of both, were considered active [27]. It means; 4) The cognitive assessment was performed using modified Mini Mental State Examination - MMSE, validated by Icaza and Albala [28]. This instrument consists of 13 questions that are independent of the school, and its cut-off scores less than or equal to twelve; 5) The depressive symptoms as assessed by the Geriatric Depression Scale(GDS) [29] validated for the Brazilian population; it was considered as a point cut scores greater than or equal to six [30]; 6) Participants who were self-rated as being unable to perform instrumental activities of daily living, or at least one ADL, without any help were defined as dependent. Activities of Daily Living (ADL): crossing fourth walking, eating, bathing, going to the bathroom, transferring from bed to chair and getting dressed. Instrumental Activities of Daily Living (IADL): using transportation, using the phone, going shopping, taking medication, taking care of one’s own money; 7) Assessment of motor function by Balance and Gait (Time Up and Go). The TUGT was assessed in the standard manner: patients were asked to rise from a 45 cm-high chair, walk forward 3 m at their usual walking pace, turn 180°, walk back to the chair and sit down. It was emphasized to participants that they undertake the test at their ‘usual walking pace’ [31]; 8) The handgrip strength was measured using a dynamometer. The test was performed in the dominant upper limb, in two attempts, which used the highest value obtained.

### Statistical analysis

The Shapiro Will test was performed to investigate whether the continuous variable gait was normally distributed. The dependent variable was not considered normally distributed, so it was stratified into quartiles according to sex and height (Table 1). This choice of stratification was based on literature because gait speed can be expected to be reduced in individuals of greater age and of lesser height, so height was considered a confusion variable that could influence the results [32]. The first quartile consisted of individuals with lower gait speed, and the fourth quartile includes faster gait speed values. For the descriptive analysis, mean and standard error values were calculated for the continuous variables, and proportions were calculated for the categorical variables. Differences between groups were estimated using the Wald test of mean equality and the Chi-Square Rao-Scott correction, which considers sample weights for estimates with population weights [33]. We adapted the significance level for the tests a p value <0.05.

**Table 1:** **Gait speed stratified into quartiles according to sex and height**

		**Men**		**Women**	
	**Quartile**	**height ≤ 1,66m**	**height > 1,66m**	**height ≤ 1,52m**	**height > 1,52m**
Gait speed (m/s)	1º quartile	≤ 0,68	≤ 0,78	≤ 0,63	≤ 0,68
	2º quartile	0,69 – 0,81	0,79 – 0,90	0,63 – 0,78	0,69 – 0,81
	3º quartile	0,82 – 0,97	0,91 – 1,04	0,79 – 0,91	0,82 – 0,95
	4º quartile	≥ 0,98	≥ 1,05	≥ 0,92	≥ 0,96

To investigate the factors associated with lower gait speed, a multinomial logistic regression was chosen. To multiple analyses, variables with p-value <0.20 were selected in the univariate analysis. In the final model, a significance level of 5%was considered. The software used was Stata ® version. The survey commands of the statistical software Stata 10 was used to analyze the data considering the complex sample design. Thus, the participants’ weight (main weight) was incorporated in the analysis.

## Results

Most elderly subjects were women (60.3%), white ethnicity (self-declared) (58.4%), had between four and seven years of study (38.1%), lived with someone else (56.7%), self-reported regular health (51%), were inactive (59.6%), had two or more chronic diseases (54.4%) and 48.3% were overweight. Prevalence values found were 7.5% for cognitive impairment and 17.6% for depressive symptoms. Regarding disability, 33% were dependent in at least one IADL and 24.7% in at least one ADL.

The average walking speed of the subjects was 0.81 m/s – 0.78 m/s among women, and 0.86 m/s among men. It was observed that gait speed decreased with older ages (p = 0.000). Among people aged 75 years or older, 46.9% and 38.7% illiterate were in the first quartile of gait speed, (p<0.001) (Table 2). Race/ethnicity had no effect on walking speed (p = 0.939). Many factors revealed significant association with lower gait speed as being illiterate (38.7%), living alone (30.0%), bad or very bad self-reported health (43%), and cognitive impairment (60.4%) (Table 3). Lower handgrip strength of the dominant hand, higher TUG, having some kind of inability, at least one ADL or IADL, and having 2 or more chronic diseases such as AVC or DCV composed the first quartile (Table 3).

**Table 2:** **Distribution (%) of older adults according to gait speed quartiles**

**Variables**		**Gait speed (%)**				**Value p**
	**Total**	**1° Q**	**2° Q**	**3° Q**	**4° Q**	
Age						0.000
60-74 years	74.9	17.6	30.8	27.5	24.0	
75 years and more	25.1	46.9	27.3	16.1	9.7	
Sex						0.987
Male	39.7	25.3	29.3	24.7	20.7	
Female	60.3	24.8	30.3	24.7	20.2	
Ethnicity						0.939
No White	41.6	25.7	29.9	24.7	19.7	
White	58.4	24.6	29.7	24.8	21.0	
Education						0.000
Illiterate	11.8	38.7	35.8	16.4	9.1	
1 to 3 years	22.4	31.4	34.3	18.0	16.3	
4 to 7 years	38.1	25.6	29.5	27.3	17.6	
8 years and more	27.7	13.0	24.5	29.9	32.6	
Conjugal situation						0.001
with someone	43.3	21.4	30.0	24.5	24.1	
Alone	56.7	30.0	29.2	25.1	15.7	
BMI						0.701
Underweight	12.5	27.7	32.1	24.5	15.2	
Eutrophic	39.2	23.9	28.3	26.0	21.8	
Overweight	48.3	25.2	30.7	23.4	20.6	
Enough income						0.044
No	42.9	18.5	22.5	34.8	24.0	
Yes	57.1	21.8	26.6	26.3	25.3	

São Paulo-SP, Brazil. SABE Study, 2010. (n = 1112).

*Abbreviations*: BMI: Body Mass Index. Income level is presented as monthly income measured in multiples of the minimum wage.

**Table 3:** **Health status and lifestyle according to gait speed of elderly in São Paulo-SP, Br**

**Variables**	**Total**	**Gait speed (%)**				**Value p**
		**1° Q**	**2° Q**	**3° Q**	**4° Q**	
Handgrip - (SE)*	25.8 (0.4)	22.9(0.7)	25.0 (0.6)	27.0(0.7)	28.6(0.9	0.000
TUG/mean (SE)**	12.9 (0.2)	17.6 (0.4)	12.6 (0.1)	11.1 (0.2)	9.7 (0.1)	0.000
Self-rated health						0.000
Very good	51.0	16.1	30.3	28.0	25.6	
Regular	42.5	28.4	30.0	25.1	16.5	
Bad/very bad	6.5	43.0	25.6	13.2	18.1	
Cognitive mpairment						0.000
Yes	7.5	60.4	29.0	3.8	6.7	
No	92.5	22.1	30.0	26.3	21.5	
Depression						0.006
Yes	17.6	28.2	36.4	21.1	14.3	
No	82.4	21.9	28.8	26.7	22.6	
Physical activity						0.070
Sedentary	59.6	28.1	29.8	23.6	18.5	
Active	40.4	20.4	30.1	26.2	23.3	
IADL disability						0.000
No	67.0	16.7	27.9	29.8	25.5	
Yes	33.0	41.3	33.9	14.5	10.4	
BADL disability						0.000
No	75.3	19.6	29.5	27.9	23.0	
Yes	24.7	41.5	31.2	14.7	12.6	
Chronic disease						0.004
None	16.8	17.6	29.7	24.8	27.9	
1	28.8	20.3	28.7	26.7	24.2	
2 or more	54.4	29.8	30.6	23.5	16.1	
Hipertension	66.4	28.6	29.6	23.2	18.6	0.003
Diabetes	25.3	27.0	30.7	23.2	19.1	0.727
COPD	9.3	24.3	44.5	22.8	8.4	0.001
DCV	22.8	32.6	31.8	24.5	11.1	0.000
AVC	3.5	58.3	23.8	10.1	7.8	0.000
Artrithis	32.3	28.9	32.5	23.4	15.2	0.012

SABE Study, 2010.

*Abbreviations*: COPD:Chronic Obstructive Pulmonary Disease, DCV:Cardiovascular Disease, AVC: Cerebrovascular Accident. SE: Standard Error *TesteWald: the means are statistically different from each other in walking speed quartiles (p <0.05) except between the means of the 1st and 2nd quartiles (p = 0.186) **TesteWald: the means are statistically different from each other in walking speed quartiles (p < 0.001).

In the final model, the factors associated with lower gait speed were being older (OR = 3.56), being illiterate (OR = 3.20), having difficulty in one or more IADL (OR = 2.74), presence of cardiovascular disease (OR = 2.15) and being active as a protection factor (Table 4). When we consider the 50% slower, we can add the variables handgrip, and presence of COPD.

**Table 4:** **Factors associated with gait speed according to quartiles of older adults**

	**1°Q**	**p**	**2° Q**	**p**	**3° Q**	**p**
**Age**						
60 to 74 y	1.00		1.00		1.00	
75 y and more	3.56	0.000	1.45	0.230	1.21	0.098
**Education**						
illiterate	3.20	0.017	2.97	0.024	1.74	0.216
1 to 3y	2.78	0.003	2.17	0.011	1.10	0.779
4 to 7 y	2.93	0.000	2.10	0.005	1.59	0.067
8 y or more	1.00		1.00		1.00	
**Handgrip**	0.98	0.154	0.97	0.023	0.98	0.362
**IADL**						
No	1.00		1.00		1.00	
Yes	2.74	0.000	1.90	0.009	1.08	0.762
**DCV**						
No	1.00		1.00		1.00	
Yes	2.15	0.006	1.82	0.037	2.05	0.017
**COPD**						
No	1.00		1.00		1.00	
Yes	1.45	0.368	2,85	0.368	2.03	0.089
**Active**						
No	1.00		1.00		1.00	
Yes	0.56	0.027	0.66	0.062	0.80	0.358

SABE Study, 2010.

*Abbreviations*: IADL: Instrumental Activities of Daily Living; ADL: Activities of Daily Living.

The model was adjusted for mini mental state examination, ADL, conjugal situation.

## Discussion

Knowing the factors associated with lower gait speed in quartiles allows us to propose actions that target each modifiable risk criterion in ageing, and reference values from healthy individuals are important for comparison to other samples and populations with different characteristics and limitations. This is the first study in a developing country with special focus on the social determinants of health showing that poor socioeconomic conditions, together with modifiable factors, play an important role in gait speed. Being older, illiterate, having difficulty in one or more instrumental activities of daily living, the presence of cardiovascular disease and being sedentary are independent factors associated with lower walking speed among the elderly.

Our results were similar to those found worldwide [4,32]; and nationally [30,31] they indicate that gait speed decreased with older age. Our older adults, however, were significantly slower than foreign populations [34,35] and their gait speed was slower than the overall fast gait speed of participants who were 70 and older with mobility limitations living in community [36]. Bohannon (2011) found that for healthy women and men aged 70–79, the usual gait speed was 1.13 m/s and 1.26 m/s, respectively, and for those aged 80–89, the values were 0.94 and 0.97 m/s respectively [36], both higher values compared to our result. The gait speed of older adults in our study were similar to the elderly aged 80–89 living in community in Dublin(Ireland), 30 percent of whom needed more assistance to walk and longer TUG, 14.2 s (versus 5.6) compared to the Brazilian sample showed in this study [37].

Older adults aged 75 or over observed in our study were 3.56 (OR) more likely to be slower compared to younger subjects, and causal factors have been widely cited in the literature such as the loss of alpha motor neurons after the seventh decade [34], the loss of type II fibres [35] and muscle mass, with more rapid decline after age 65 [36,37] and the interposition of fat in muscle decreases muscle contraction [10].

Interestingly, comparing our results with those obtained from another sample of Brazilian elderly subjects – *FIBRA* network study (Frailty among Brazilian Older Adults), our average gait speed value was slower than the average of (1.11 m/s) that study. The *FIBRA* study included subjects from different Brazilian cities with different Human Development Indexes, at an increased average age of 71.4 [34,35,37,38]. Besides that, the percentage of illiterate older adults was smaller and the methodology was different from our research. Unlike the *FIBRA* study, which used a convenience sample, ours used a weigthing sample in which a weight is attributed to each individual, which indeed makes it a representative sample of the city of Sao Paulo. Other studies included volunteer subjects [33,32] or only women [34].

Maybe these differences can explain why independent associations between gait speed and educational level or income were observed only in our study. Another relevant point: our data indicate a population of elderly patients living in São Paulo with chronic diseases that affect mobility and result in low physical activity [38]. According to international literature, our older adults are deemed as frail and [39] at high risk of poor outcome [10] and poor survival [8].

Despite gait assessment being a quick, safe, inexpensive and highly reliable measure, methodology can vary widely making it difficult to compare studies. Similar methods can differ in walking length, and different populations weaken the comparisons.

Although in Studenski’s research (2011) the vast majority of the sample comprised white men and women, similar characteristics observed in our study, the mean gait speed in older adults was 0.92 m/s [8], a higher value compared to our study despite 45% of all participants being older than 85. Watson [6], analyzing data from well-functioning sub-cohort (USA), found mean gait speed of 1.20 m/s and the mean gait speed of the first quartile slower than 1.05 m/s; a higher value comparing to our results although mean age of sample was higher (75.2 years) Although the subjects in Watson’s studies were older, and 68.2% of them were sedentary, they were faster. A factor that may have influenced his study more favourably is that his sample comprised greater numbers of men and black individuals; moreover, those subjects had more education than the ones in our sample. However, similarly to our results, the participants in the lower quartile of gait speed were more likely to be older, sedentary, have less education and have more chronic health conditions.

Our results revealed that gait speed increases with the highest level of education (OR 3.20), similar to that found in Brunner’s study (2009) [40]. Years of schooling are used as a proxy for social status and, thus, health condition. Most of the elderly individuals in Brazil live in poor conditions, particularly in São Paulo, a city with great economic and social contrasts (almost 40% of the elderly subjects were illiterate). Although education alone does not ensure the end of social discrimination, it is part of the formation of a more egalitarian society and a critical factor in reducing socioeconomic disparities. Despite the positive correlation between education and income, education is considered a major factor in overcoming income inequality. Educational level is a protective factor and prevents poor outcomes in health. Individuals with more education are more likely to obtain financial resources, seek medical advice and detect diseases earlier; therefore, they have better self-reported health, get better health treatment and better understand the importance of prevention, such as doing physical activity and, thereby, decrease their chance of comorbidity. It is a fact that prevalence of chronic disease may also be influenced by an individual’s access to health services, by their socioeconomic condition, and self-reported health status [41]. Self-reported health is recognized internationally as an indicator of health status, and may justify a positive association with gait speed in bivariate analyses.

Although a positive correlation between handgrip and gait speed was observed, the handgrip association did not remain significant to the first quartile of gait speed in the final model, probably due to the fact that the subjects were already very committed in walking speed.

Regarding the TUG, our study found a mean of 12.9 s, a higher value than the results found in an meta-analysis (9.4 s) [39]. Although we did not explore the mechanisms underlying the changes observed in this study, the negative correlation found between TUG and gait speed shows the importance of evaluation of gait and balance. Gait is dependent on postural control and the integration of various systems, such as proprioceptive, visual and vestibular, their sensory input, integration in the Central Nervous System (CNS) and, depending on effective motor response [39,40] and gait assessment, it is a form of prevention against disability and motor decline [16].

Another population-based study revealed that each increment of one standard deviation in the usual gait speed was associated with a reduced likelihood of disability from 26 to 44% [41]. Similarly, our current findings revealed 33.7% of subjects disabled in one or more instrumental activities with 2.74(OR) to be on the first quartile of gait speed.

The results presented here show in bivariate analysis that the presence of cognitive impairment was significantly associated with gait speed. The same results were found in other studies, reinforcing the notion that cognition influences gait speed [6,42,43]. The significant association between gait and cognition maybe can be explained by the influence of cognitive aspects and mood on the maintenance of functional capacity, and by the need of physical and intellectual integrity to remain autonomous and independent. Although it is discussed whether cognitive decline is a predisposing or precipitating factor in the decline of gait speed [44], our data seems to indicate that the decline in physical function is secondary to cognition. Perhaps most of the older adults in São Paulo cannot afford cognitive rehabilitation services.

Regarding depression, the present results show that depression levels have a positive correlation to gait speed, which agrees with what Mossey and colleagues presented (2000) [45]. Adopting a healthier lifestyle is an important part of treating depression, e.g. doing physical activities on a regular basis. Previously published systematic reviews and meta-analysis concluded that exercise reduces depressive symptoms among patients with a chronic disease [46,47]. Research has also shown that depressed patients are less fit and have diminished physical work capacity [48], which in turn may contribute to other physical health problems. Depression in Brazil is underdiagnosed, probably because its diagnosis is often hampered by the presence of comorbidities, the difficulty of the healthcare teams to recognize it and the lack of mental health care in the primary health care system. Studies show that between 50 - 60% of the cases of depression are not detected or adequately treated [49]. Furthermore, depending on the intensity of the depressive symptoms, it becomes impossible to motivate the subject to do physical activity.

In the final model, those who considered being active showed to be significantly associated with higher walking speed. One of the important ways to prevent the insidious loss of bone and muscle strength is to stay active. When an individual loses muscle strength, walking becomes less frequent and slower, as one becomes physically unconditioned. Consequently, the individual becomes more sensitive to fatigue and, thereby, increases inactivity. Once this vicious cycle is triggered, it ends up compromising initially instrumental activities and, subsequently, the basic ones if nothing is done to halt the cycle. Physical inactivity is an important risk to cardiovascular disease. It was shown, however, that it is preventable, up to 80%, by eliminating shared risk factors such as physical inactivity [50]. Our data revealed an important association between cardiovascular diseases with the lowest quartile of gait speed in the final model. This reinforces the fact that the usual speed of gait is related to aerobic capacity showing association with functional reserve [51]. Walking imposes demands on the nervous, cardiovascular, pulmonary, musculoskeletal and hematologic systems, as they require more oxygen to contract the muscles. These systems work synergistically – if one of them does not work well, it can impair gait speed [8].

Other studies showed that regular exercise significantly improved physical fitness (aerobic capacity), walking capacity and cardiovascular dimensions [52].

Considering the relevance of this problem, Matsudo and colleagues interviewed 2001 individuals aged 14–77 in 29 cities within the state of Sao Paulo and showed that the levels of physical activity did not differ among age ranges. There were similarities between the genders, but people from metropolitan regions and the poorer ones were less active [53]. Probably the modern world with electronic novelties encourages a sedentary life style.

Another study conducted in Santos (a beach city in Brazil) recruited healthy elders of both genders as volunteers, who also led a sedentary life style. Their gait speed was 1.34 m/s among men, and 1.27 m/s among women. The values of gait speed found were significantly lower than those foreign benchmarks (p < 0.05) [32,54] but higher than our findings in São Paulo, probably because of different habits and socioeconomic backgrounds. It is important to mention that although our data has been adjusted for height, the average height of the Brazilian elderly population is shorter compared to populations of a similar age range from developed countries.

Regarding cerebrovascular disease and the lack of association with lower walking speed in the final model, it could be explained by the exclusion of all individuals with motor sequels, which could have influenced gait measures. COPD is a systemic disease that affects beyond the respiratory, cardiovascular and muscular systems. Among the muscle changes are loss of muscle mass, loss of efficiency to carry out protein synthesis, decrease in type I fibres [55,56]. There are several factors that can cause these changes in the muscular system such as chronic hypoxemia, prolonged usage of high doses of corticosteroids, nutritional changes, the response to systemic inflammation and even physical deconditioning [56]. The inability to exercise and the ventilatory limitations increase deconditioning, which ends up compromising their functionality [55]. These factors may explain the significant association between lower gait and COPD found in the bivariate analyses. However, this association was not found in the multivariate analyses. Although 54.4% of the elderly subjects reported two or more chronic diseases, 50.9% related their health as very good, which reinforces that health is no longer measured by the presence or absence of diseases, but by the degree of preservation of one’s functional capacity and independence. Such result was also evidenced by the high prevalence of elderly people without disability in basic and instrumental activities. What is at stake in old age is autonomy, the ability of the elderly to remain socially integrated and, for all purposes, health [57]. In our study, 46% of the elderly aged 75 years or over were in the first quartile, which could be related to the prevalence of more chronic diseases and worse handgrip strength; they had lower educational levels and were inactive, similar to other studies [6,9].

### Study limitations

Despite the advantages of quickness and costs of the cross-sectional study, it presents limitations since it does not allow one to identify causality, whether the factors identified as associated to lower gait speed came before or after it, since expositions and outcomes are collected at the same moment. This may also explain the lack of significance between race and lower gait speed found in this study. In addition, the presence of chronic diseases, health condition, disability and physical activity were assessed by means of self -reporting, which may result in over or under-estimation of prevalence. However, participants report only those conditions diagnosed by a physician. As to physical activity, IPAQ was validated in a sample of the Brazilian population. Older adults who used assistive devices to walk, or those with severe neurological conditions, were excluded, which might limit the external validity of the study. Our current results show that poor socioeconomic conditions present in developing countries influence lower walking speed such as education, and they may be particularly related to some modifiable factors such as impairment of IADL, physical inactivity and cardiovascular disease.

## Conclusion

The current results revealed an elderly population with a lower average speed compared to older adults from developed countries. A population-based cross sectional study reflected the exact condition of the elderly. It suggested that biological and socioeconomic factors such as one’s education level and lifestyle might interfere in one’s health condition, as it may also explain the different patterns of gait speed.

The identification of the factors related to lower walking speed is essential to elaborate preventive actions to be carried out before senior citizens reach worse gait speed, which can be a proxy for other conditions that lead to an undesirable health outcome. These results alert for the prevention of CVD avoiding lower gait speed and impaired functional capacity, thus reinforcing the importance for the elderly to remain active and age healthy.
